# Environmentally Relevant Dose of Bisphenol A Does Not Affect Lipid Metabolism and Has No Synergetic or Antagonistic Effects on Genistein’s Beneficial Roles on Lipid Metabolism

**DOI:** 10.1371/journal.pone.0155352

**Published:** 2016-05-12

**Authors:** Shibin Ding, Xuezhi Zuo, Ying Fan, Hongyu Li, Nana Zhao, Huiqin Yang, Xiaolei Ye, Dongliang He, Hui Yang, Xin Jin, Chong Tian, Chenjiang Ying

**Affiliations:** 1 Department of Nutrition and Food Hygiene, School of Public Health, Tongji Medical College, Huazhong University of Science and Technology, 13 Hangkong Road, Wuhan, 430030, PR China; 2 Department of Nutrition and Food Hygiene, School of Public Health, Xinxiang Medical University, Xinxiang, 453003, PR China; 3 Department of Clinical Nutrition, Tongji Hospital, Huazhong University of Science & Technology, Wuhan, 430030, PR China; 4 Department of Psychiatry, The Fifth Hospital of Xiamen, Xiamen, 361101, PR China; 5 Department of Nosocomial Infection Management, Central Hospital of Suizhou, Suizhou, 441300, PR China; 6 School of Environmental Science and Public Health, Wenzhou Medical University, Wenzhou, 325000, PR China; 7 Department of Nursing, Tongji Medical College, Huazhong University of Science & Technology, Wuhan, 430030, PR China; 8 MOE Key Lab of Environment and Health, School of Public Health, Tongji Medical College, Huazhong University of Science and Technology, 13 Hangkong Road, Wuhan, 430030, PR China; University of Missouri, UNITED STATES

## Abstract

Both bisphenol A (BPA, an endocrine disrupting chemicals) and genistein (a phytoestrogen mainly derived from leguminosae) are able to bind to estrogen receptors, but they are considered to have different effects on metabolic syndrome, surprisingly. We here investigate the effects of an environmentally relevant dose of BPA alone and the combined effects with genistein on lipid metabolism in rats. Eight groups of adult male Wistar rats, fed with either standard chow diet or high-fat diet, were treated with BPA (50μg/kg/day), genistein (10mg/kg/day), and BPA plus genistein for 35 weeks, respectively. Metabolic parameters in serum and liver were determined; the hematoxylin/eosin and oil Red O staining were used to observe liver histologically; gene expressions related to hepatic lipid metabolism were analyzed by Real-time PCR; protein expressions of PPARγ, PPARα and LC3 in liver were analyzed by western blotting. No difference of body weight gain, total energy intake, liver weight/body weight or body fat percentage in both STD- and HFD-fed sub-groups was observed after treatment with BPA, genistein, or BPA plus genistein (*P*>0.05). Genistein alleviated lipid metabolism disorder and decreased the mRNA and protein expression of PPARγ (*P*<0.05), and increased the protein expression of LC3II (*P*<0.05) in liver of HFD-fed rats. However, BPA treatment had no effect on lipid metabolism in rats alone (*P*>0.05) or combined with genistein. Our findings suggest that long-term environmentally relevant dose of BPA did not affect lipid metabolism, and had no synergetic or antagonistic roles on genistein’s beneficial function on hepatic lipid metabolism.

## Introduction

The prevalence of the metabolic syndrome (MetS) has dramatically increased worldwide and is now recognized as a serious public health problem. MetS represents a cluster metabolic abnormality including abnormal elevated glucose level, central obesity, hypertension, dyslipidemia, insulin resistant [1], and non-alcohol fatty liver disease (NAFLD) is now considered to be the hepatic component of the MetS [2,3]. There are multiple factors influencing the initiation and development of MetS, including those from diet, environment and endocrine. Bisphenol A (BPA) is an endocrine disrupting chemicals (EDCs) and monomer of polycarbonate, which is widely used in the synthesis of polycarbonates plastics and epoxy resins for many years. Because of the widespread applications in the manufacture of plastic food or beverage containers and the protective coatings of metal cans, people are commonly exposed to environmental relevant dose of BPA. Thus, BPA exposure has received increased concern regarding the adverse health effects. The dose of BPA less than 50μg/kg/day (tolerable daily intake, TDI) is considered as “safe” established by the U.S. Environmental Protection Agency (EPA) and the European Food Safety Agency [4]. However, some studies have demonstrated that treatment with BPA (10 to 50μg/kg/day) increased insulin secretion in swiss albino OF1 male mice (8–10 weeks old) [5] and aggravated the insulin resistance of female swiss albino OF1 mice produced during pregnancy [6], and long-term BPA-exposure disrupted glucose homeostasis of Wistar rats in our previous study [7]. These animal studies indicated that the predicted “safe” dose may not be really safe. Grasselli *et al*. [8] also reported that BPA (30 and 300 ng/mL) has a direct action on lipid homeostasis of hepatoma cells in rat which was deemed to interfere with lipid oxidation and secretion. Moreover, different doses of BPA (5, 50, 500, and 5,000μg/kg/day) exposure for 28 days may affect de novo fatty acid synthesis of liver through increased expression of lipogenic genes in six-week-old male CD1 mice [9]. Therefore, the risk of environmentally relevant doses of BPA on hepatic lipid metabolism needs to be further assessed.

Since 3 decades ago, soy consumption has been on the rise in western cultures and diets [10]. Additionally, soy-based infant formulas has been used in Western infant formulas for western countries for more than 40 years, infants consumed isoflavones (6–11 mg/kg/day) from soy-based formulas [11]. Genistein (4’,5,7-trihydroxyisoflavone), a phytoestrogen and main component of isoflavones, is ubiquitous in many plants which are usually consumed by people and animals, and is especially abundant in leguminosae. Thus, people over the world have frequently consumed different levels of genistein sources primarily from soy foods. Recently, genistein has gained attention for beneficial effects on MetS and NAFLD in rodent and human studies [12–14], which has been suggested to prevent these diseases. However, in these rodent studies, the doses of genistein treatment which play a protective role in glucose and lipid metabolism is considerably higher than the dose of genistein as a daily dietary consumption in Asian (“dietary dose”, 6mg/kg/day) assessed Japanese women [15], and is also higher than no observed adverse effect level (NOAEL) of genistein (50 mg/kg/day) observed in acute, subchronic and chronic safety studies of Wistar rats [16]. Therefore, it is still unclear whether long-term genistein treatment could have beneficial effects on lipids metabolism.

Due to the widely used of BPA in the industrial sector, both Eastern and Western populations were exposed to BPA at an environmental relevant level in life; meanwhile, both Eastern and Western populations take genistein through soybeans consumption or supplementation daily. Therefore, under an environmental relevant dose of BPA exposure, whether genistein could still play a beneficial role on lipid metabolism of Eastern and Western populations remains unclear. In our study, the effects of alone and combined treatment with environmentally relevant dose of BPA and genistein on lipid metabolism were studied in adult male rats fed with STD (represents Eastern diet) or HFD (represents Western diet). Oral intake is the most common route for genistein and BPA. The result of treatment via oral are fit to be extrapolated to humans. To avoid the effects of estrogen and menstrual cycle in female rats, the male rat was chose in our study. As autophagy, a process involved in the degradation of unnecessary cellular components and rearrangement of subcellular membranes to sequester cytoplasm and organelles in all eukaryotic cells [17,18], had been recently shown regulating lipid metabolism via involving triglycerides and lipid droplets breakdown [19]. We observed upregulated expression of microtubule-associated protein 1A/1B-light chain 3 (LC3) protein (autophagy marker) in the pancreatic β-cell of rats in the HFD-treated and BPA-treated groups [7]. Exploring the effects of BPA and genistein on both classic and autophagy mechanism of lipid metabolism will be of interest.

To the best of our knowledge, such detailed analysis exploring the effects of long-term environmentally relevant dose of BPA treatment alone and combined with genistein on liver lipid metabolism in adult male rats has not been reported.

## Materials and Methods

### Chemicals or reagents

Genistein (purity>98%) was obtained from Fuzhou Rimian Inc. (Fuzhou, FuJian, China); and Bisphenol A (BPA), (CAS No.80-05-7, (CH_3_)_2_C(C_6_H_4_OH)_2_, ≥99% purity) was obtained from Sigma Chemical Company (St. Louis, Missouri). Assay kits for serum total cholesterol (TC), and triglycerides (TG) were purchased from BIOSINO Biotechnology and Science Inc. (Beijing, China). TRIZOL was obtained from Invitrogen Inc. (Carlsbad, CA, USA) and Real time quantitative PCR kit was purchased from TAKARA Bio Inc. (Otsu, Shiga, Japan). Insulin antibody was obtained from Merck Millipore (Billerica, MA, USA). Rabbit anti-LC3B was purchased by Cell Signaling Technology (Billerica, MA, USA), PPARα and PPARγ antibody were obtained from Abcam (Cambridge, UK). All other chemicals were of the highest grade commercially available.

### Animal treatment

Animal procedures performed in the study had been approved by the Animal Care and Ethical Use Committee of Tongji Medical Collage, in accordance with the guidelines for care and use animals established by Tongji Medical College, Huazhong University of Science and Technology.

Male 8-week-old Wistar rats (150–180g) were supplied by the Hubei Research Center of Laboratory Animal, China. Animals were provided with standard rodent chow and tap water *ad libitum* and housed at 21±1°C, 60±10%-relative humidity, while on a regular 12 h light/dark cycle. Animals were kept in polypropylene cages.

### Experimental design

After one week acclimation, all rats were randomly divided into 8 groups with 10 rats per group and treated for 35 weeks as follows: (1) STD group was fed with rodent standard chow diet (STD); (2) STD-BPA group was fed with STD and administered with BPA (50μg/kg/day); (3) STD-(BPA+G) group was fed with STD and administered with BPA (50μg/kg/day) plus genistein (10mg/kg/day); (4) STD-G group was fed with STD and administered with genistein (10mg/kg/day); (5) HFD group received high-fat diet (HFD); (6) HFD-BPA group was fed with HFD and administered with BPA (50μg/kg/day); (7) STD-(BPA+G) group was fed with HFD and administered with BPA (50μg/kg/day) plus genistein (10mg/kg/day); (8) HFD-G group was fed with HFD and administrated with genistein (10mg/kg/day). All the male genitors were treated for 35 weeks consecutively. The details of BPA (50μg/kg/day) and genistein (10mg/kg/day) treatment methods have been described previously: BPA was dissolved in corn oil and diluted with three stock solutions (20, 40, 80, and 120 μg/ml), genistein was dissolved in corn oil and diluted with three stock solutions (10, 20 and 30 mg/mL), and were store at -20°C. The necessary volumes of stock solution were calculated based on the daily body weight and the suitable stock solution was selected to limit the volume of stock solution less than 0.5 ml. Corn oil was added to this solution to form a total volume of 0.5 ml, this was then directly mixed with standard rodent chow (5 g/rat) as the test diet. The energy supply of two diets is listed in Table 1. The composition of high-fat diet is as we previously described [20]. The energy supply of two diets is listed in Table 1. The consumption of different diets was calculated daily and body weight was monitored weekly.

**Table 1:** The percent composition of main components in standard and high-fat diets.

Diets	Fat(%)	Carbohydrate(%)	Protein (%)	Total energy(KJ/g)
Standard diet (STD)	13.68	64.44	21.88	13.77
High-fat diet (HFD)	41.26	39.61	19.13	19.21

After the treatment of 35 weeks, all rats were decapitated swiftly after an overnight fasting. Serum was separated from the blood by centrifuge at 1000 g for 10 min at 4°C (Eppendorf 5810R, Hamburg, Germany). The liver and the subcutaneous fat pad were dissected and weighed were rapidly frozen in liquid nitrogen and stored at -80° for future experiments. The liver weight was used to calculate the liver weight/body weight (liver weight × 100/body weight). Subcutaneous body fat percentage was calculated by following equation: subcutaneous fat pad weight × 100/body weight.

### Energy intake assessment

Total diet consumption of each rat during 35 weeks was calculated according to daily food consumption. Total energy intake of per cage (two rats) is calculated as follows: the total consumption diet of 35 weeks multiply the percentage of fat, protein, and carbohydrate in two diet, and further multiply corresponding caloric values of fat, protein, and carbohydrate, respectively.

### Haematoxylin and eosin and oil Red O staining of liver tissue sections

To assess hepatic histology, the haematoxylin and eosin-stained of liver tissue sections was used. Paraformaldehyde-fixed fresh liver tissues from central zone of medial lobe were embedded in paraffin, and sliced into 3-μm sections. Sections were stained with hematoxylin and eosin, then coverslipped with mountant for observation. Liver tissue sections (10 mm) were fixed with paraformaldehyde, stained with Oil Red O, washed and counterstained with haematoxylin. Images acquisition was obtained by Olympus BX50 light microscope with HMIAS-2000 medical imaging system.

### Determination of serum and hepatic lipid parameters

Whole blood was collected from 12 h fasted rats and was used to separate serum by centrifugation. Serum glucose levels were measured with glucose oxidase-peroxidase assays (BIOSINO Biotechnology and Science Inc., Beijing, China). Serum TG and TC were assayed using enzymatic colorimetric assays (BIOSINO Biotechnology and Science Inc., Beijing, China) according to the manufacturer’s protocol. Hepatic lipids were extracted according to the procedure previously described [21], and the cholesterol and triglyceride concentration in the extracted lipid were determined by enzymatic kits following instruction. Serum insulin was measured using enzyme-linked immunosorbent assay kit (Bai Wo, Shanghai, China). All serum parameters and hepatic lipids were determined spectrophotometrically by SpectraMax M2 microplate reader (Molecular Devices, Sunnyvale, CA).

### Real-Time Polymerase Chain Reactions

Real-time PCR was used to detect the mRNA expression levels of LC3, the transcription factor EB (TFEB), the sterol regulatory element binding protein 1 (SREBP1C), the sterol regulatory element binding protein-2 (SREBP2), peroxisome proliferator-activated receptor-alpha (*PPARα*) and peroxisome proliferator-activated receptor γ (PPARγ), the key genes to regulate lipid metabolism in liver. The sequences are listed in Table 2. Briefly, total RNA from the liver was extracted by TRIZOL according to the manufacturer instructions then quantified by UV spectrophotometry. Total RNA samples with the value of A260/280 between 1.8 and 2.0 were used. Reverse transcription reaction (RT) of total RNA in each sample was performed according to the manufacturer’s instructions. The qRT-PCR was carried out in triplicate for target mRNAs of each sample by qPCR SYBR Green mix and specific oligo primers with the following parameters: 1 cycle, 95°, 5 s; 40 cycles, 95°C 10s; 57°C, 30 s. The fluorescent signals of SYBR Green for each amplicon were detected by ABI 7900HT machine (Applied Biosystems, Forster, CA, USA). Melting curve analyses was used to assess the specificity of the product. The 2^-ΔΔCT^ method was adopted for the data analysis and β-actin was as reference.

**Table 2:** Sequences of oligonucleotide primers used in real-time PCR.

Gene	Forward primer (5`-3`)	Reverse primer (5`-3`)	Accession No.
*LC3*	ATAGAGCGATACAAGGGTG	AGGAAGAAGGCTTGGTTA	NM_022867
*TFEB*	GCTTCCCTGGTAGGTGTCA	CTTTCTTCTGCCGTTCCTT	NM_ 001025707
*PPARα*	GAAAGATTCGGAAACTGC	TCCTGCGAGTATGACCC	NM_013196
*PPARγ*	TACCACGGTTGATTTCTC	TCTACTTTGATCGCACTTT	NM_013124
*SREBP1C*	CGACTACATCCGCTTCTTACA	AGGTTTCATGCCCTCCATA	NM_001276708
*SREBP2*	CGCCTGGTACGCTGGTTACTC	GCATCCCTCGCACTGCTCTTA	NM_001033694
*β-actin*	CGTGCGTGACATTAAAGAG	TTGCCGATAGTGATGACCT	NM_031144

LC3, microtubule-associated protein 1A/1B-light chain 3; TFEB, the transcription factor EB; PPARα, peroxisome proliferator-activated receptor-alpha; PPARγ, peroxisome proliferator-activated receptor γ; SREBP1C, the sterol regulatory element binding protein 1; SREBP2, the sterol regulatory element binding protein-2.

### Protein extraction and western blot analysis

Protein expressions of PPARα, PPARγ and LC3 in liver were measured with Western blot analysis. Rat liver extracts were prepared by homogenizing the tissue in a lysis buffer (50 mmol/L Tris/HCl (pH 8.0), 150 mmol/L NaCl, 1% Nonidet-P40, 1% sodium deoxycholate, 0.1% sodium dodecyl sulfate (SDS), 0.1 mmol/L DTT, 0.05 mmol/L PMSF, 0.002 mg/ml aprotinin, 0.002 mg/ml leupeptin, and 1 mmol/L NaVO_3_). The protein concentration was quantified with BIO-RAD DC Protein Assay Reagent (Bio-Rad, Hercules, CA, USA). Proteins were separated by sodium dodecyl sulfate-polyacrylamide gel electrophoresis and transferred to PVDF membranes according to the method of Amersham Biosciences. The blots were incubated with primary antibodies overnight at 4°C. β-actin (Sigma–Aldrich Chemical Company, St. Louis, Missouri) was used as a loading control. LC3-II/β-actin is used as an index of autophagy activity. Protein expression was visualized with an ECL detection system (Syngen, Cambridge, UK) and analyzed by software Chemidoc-Quantity-One (Bio-Rad Laboratories).

### Statistical analysis

All data were analyzed with SPSS13.0 (SPSS, Chicago, IL). Results are expressed as mean ± SEM. The body weight gain, body fat percentage, liver weight/body weight, total energy intake, total triglycerides, total cholesterol, hepatic TG and TC, as well as the mRNA expressions of *LC3*, *TFEB*, *SREBP1C*, *SREBP2*, *PPARα* and *PPARγ*, the protein expressions of LC3, PPARα and PPARγ were analyzed for statistical significance by the non-repeated ANOVA, followed by post hoc analysis (Bonferroni’s test). Statistical differences between two groups were calculated by the unpaired Student’s t test. All P values were two-tailed and P values less than 0.05 were considered significant.

## Results

### Body weight, total energy intake, liver weight/body weight and subcutaneous body fat percentage

**Fig 1:**
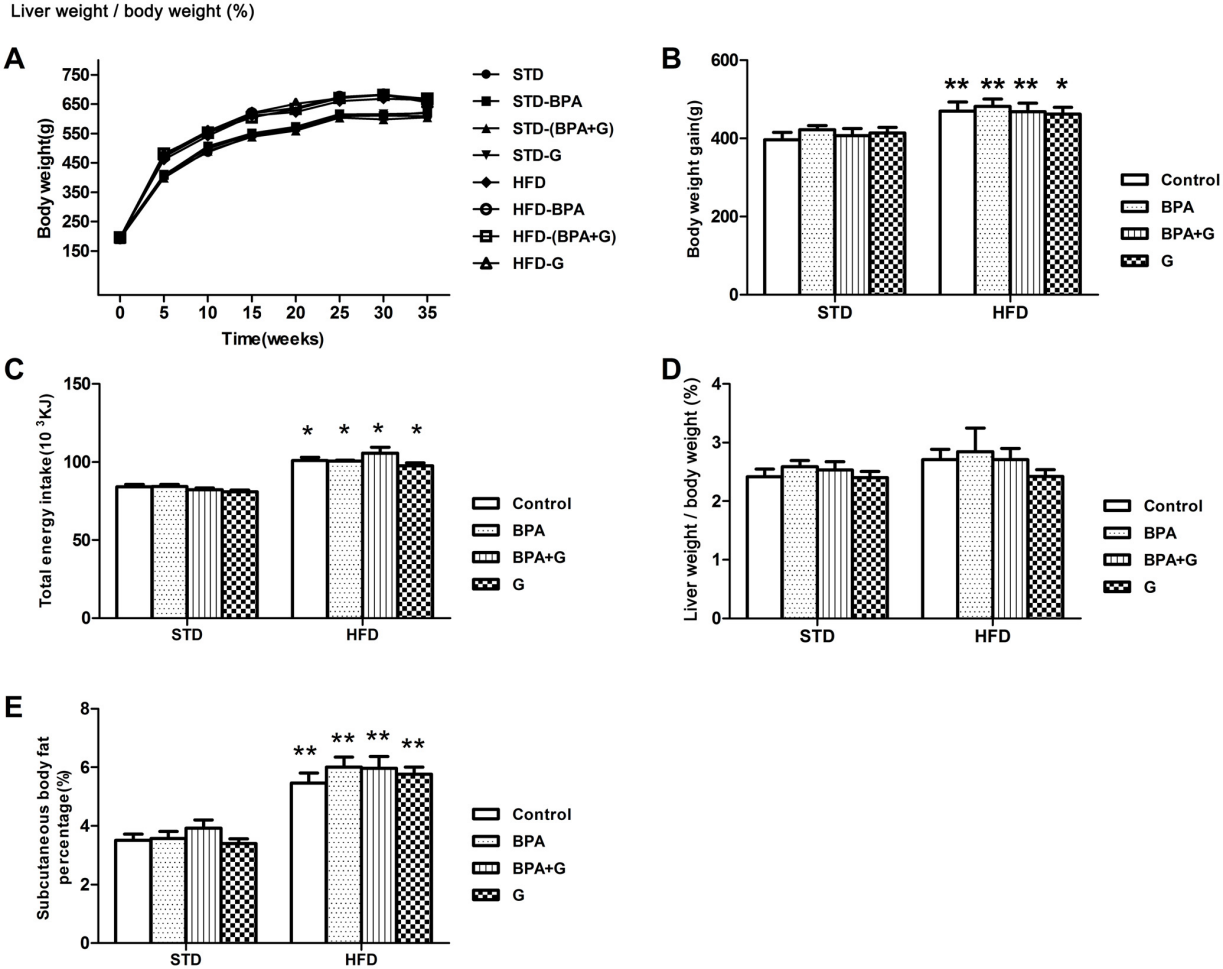
Effects of BPA and genistein on body weight, total energy intake, liver weight/body weight and Subcutaneous body fat percentage in rats. (A)Body weight (n = 8–10). (B) Body weight gain (n = 8–10). (C) Total energy intake (n = 5). (D) liver weight/body weight (n = 8–10). (E) Subcutaneous body fat percentage (n = 8–10). Data are expressed as Mean ± SEM. * *P*<0.05; ** *P*<0.01 compared to STD group.

As shown in Fig 1(Data in S1–S5 Tables), total energy intake and body fat percentage were significantly increased in HFD-fed groups compared to STD-fed groups (*P*<0.05, *P*<0.01). Significant differences of body weight gain were observed between HFD-fed groups and STD-fed groups (*P*<0.05 or *P*<0.01); but no difference of liver weight/body weight was observed between HFD-fed groups and STD-fed groups (*P*>0.05). In both STD- and HFD-fed sub-groups, treatment with BPA or genistein did not affect body weight gain, total energy intake, liver weight/body weight or subcutaneous body fat percentage of rats (*P*>0.05). Furthermore, the combined treatment of BPA and genistein also had no effect on these parameters in STD- or HFD-fed groups (*P*>0.05).

### Serum lipid and hepatic lipid parameters

**Fig 2:**
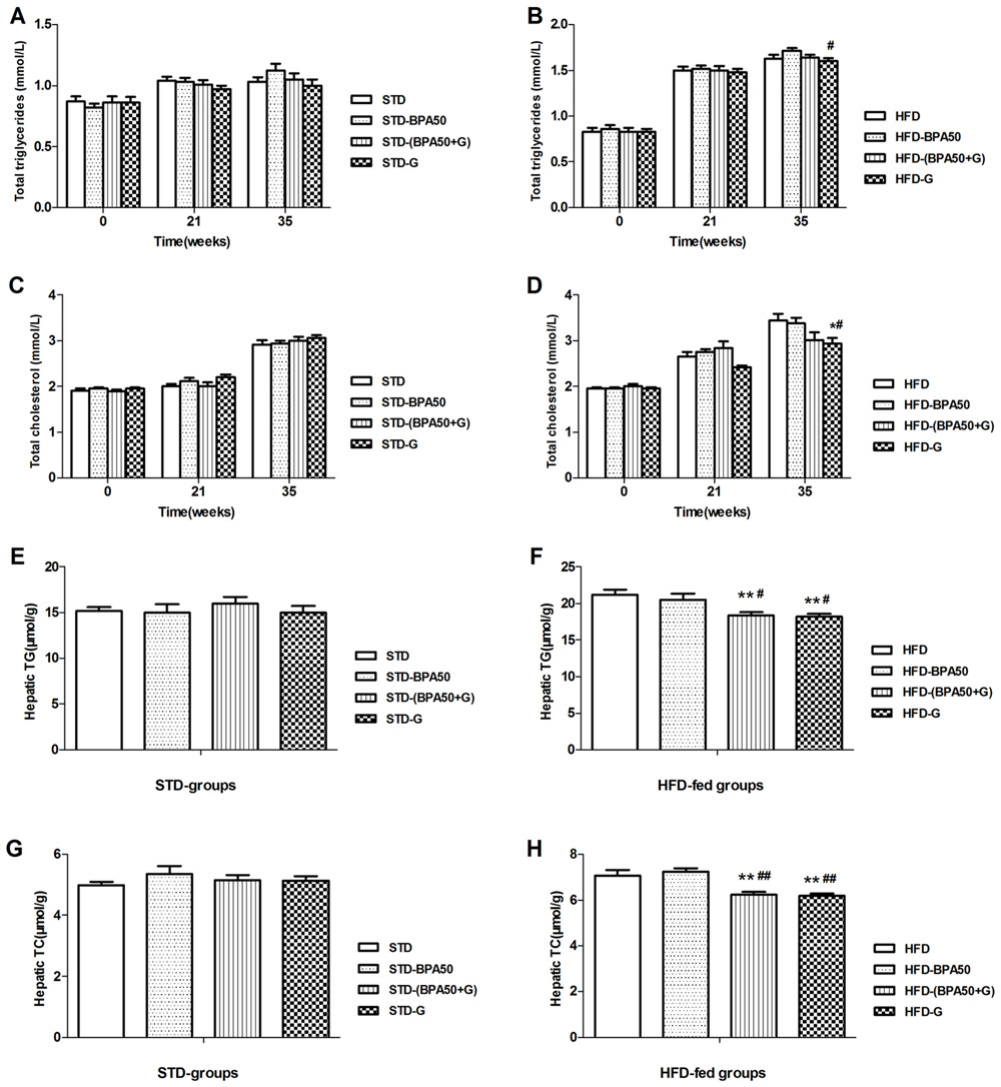
Effects of BPA and genistein on serum metabolite and liver lipid levels in rats. (A) Total triglycerides in STD fed groups. (B) Total triglycerides in HFD fed groups. (C) Total cholesterol in STD fed groups. (D) Total cholesterol in HFD fed groups. (E) Hepatic total triglycerides in STD fed groups. (F) Hepatic total triglycerides in HFD fed groups. (G) Hepatic total cholesterol in STD fed groups. (H) Hepatic total cholesterol in HFD fed groups (n = 10). Data are expressed as Mean ± SEM. * *P*<0.05, ** *P*<0.01 compared to HFD group; ^#^ *P*< 0.05, ^##^ *P*<0.01 compared to HFD-BPA group.

After 35 weeks treatment, compared to the HFD-BPA group, the TG level was significantly decreased in HFD-G group (*P*<0.05) (Fig 2B, data in S7 Table). As shown in Fig 2D (Data in S9 Table), the level of TC in HFD-G group was lower than HFD group (*P*<0.05) and HFD-BPA group (*P*<0.05). Compared to HFD group and HFD-BPA group, hepatic TG level was markedly decreased in HFD-(BPA+G) group (*P*<0.01 and *P*<0.05), as well as in HFD-G group (*P*<0.01 and *P*<0.05) (Fig 2F, data in S11 Table). Moreover, as shown in Fig 2F the hepatic TC levels in HFD-(BPA+G) group and HFD-G group were lower than HFD group and HFD-BPA group (*P*<0.01 and *P*<0.01). There were no effects of BPA and genistein treatment on TG and TC in serum and liver of STD-fed groups (Fig 2A, 2C, 2E and 2G; data in S6, S8, S10 and S12 Tables).

### Morphology and lipid accumulation of liver

**Fig 3:**
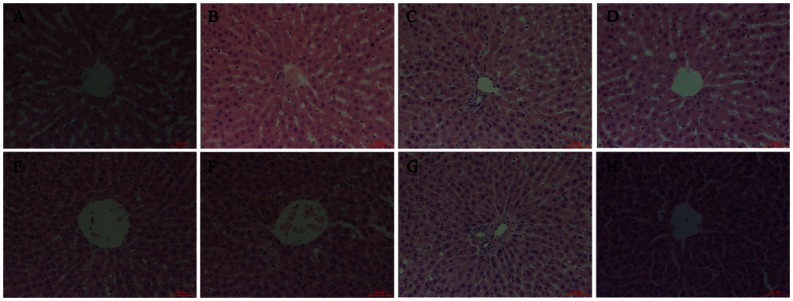
Morphological examination by hematoxylin and eosin staining of liver tissue sections in STD and HFD fed rats. (A) STD, (B) STD-BPA, (C) STD-(BPA+G), (D) STD-G, (E) HFD, (F) HFD-(BPA+G), (G) HFD-G (400×magnification, Bar = 100 μm).

**Fig 4:**
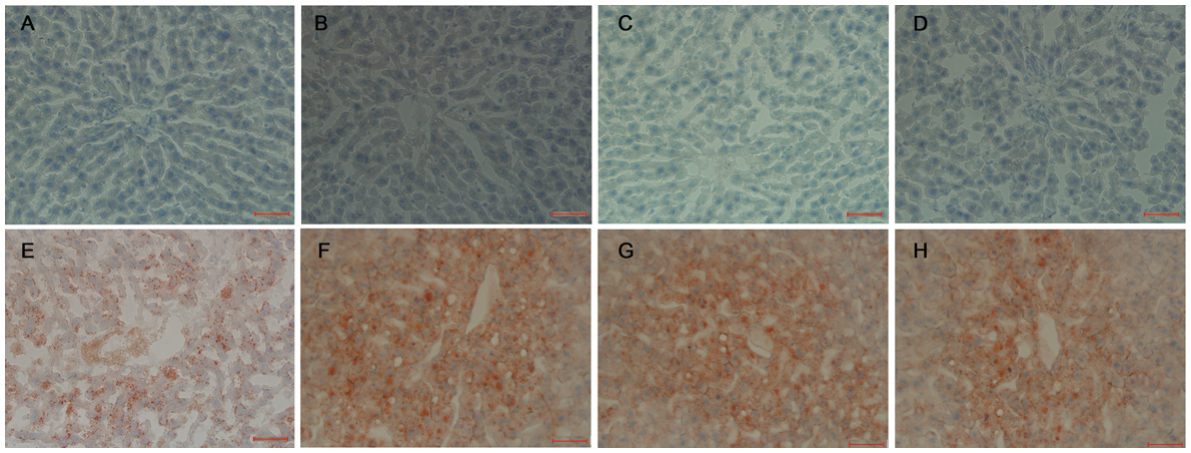
Morphological examination by oil Red O staining of liver tissue sections in STD- and HFD-fed rats. (A) STD, (B) STD-BPA, (C) STD-(BPA+G), (D) STD-G, (E) HFD, (F) HFD-(BPA+G), (G) HFD-G (400×magnification, Bar = 100 μm).

As shown in Fig 3, long-term HFD feeding resulted in a pervasive macrovesicular infiltration of lipid droplets by hematoxylin–eosin staining in rats. Genistein treatment alleviated HFD-induced morphological change of lipid droplets deposition, especially on the hepatic cells distant from the central hepatic lobule. The hepatic lipid accumulation was shown in Fig 4, compared with the STD-fed rats, HFD-fed rats exhibited a marked increase in overall hepatic lipid accumulation. The hepatic lipid accumulation was dramatically reduced in HFD-fed rats by genistein treatment (Fig 4G and 4H).

### The mRNA expression of liver in rats

**Fig 5:**
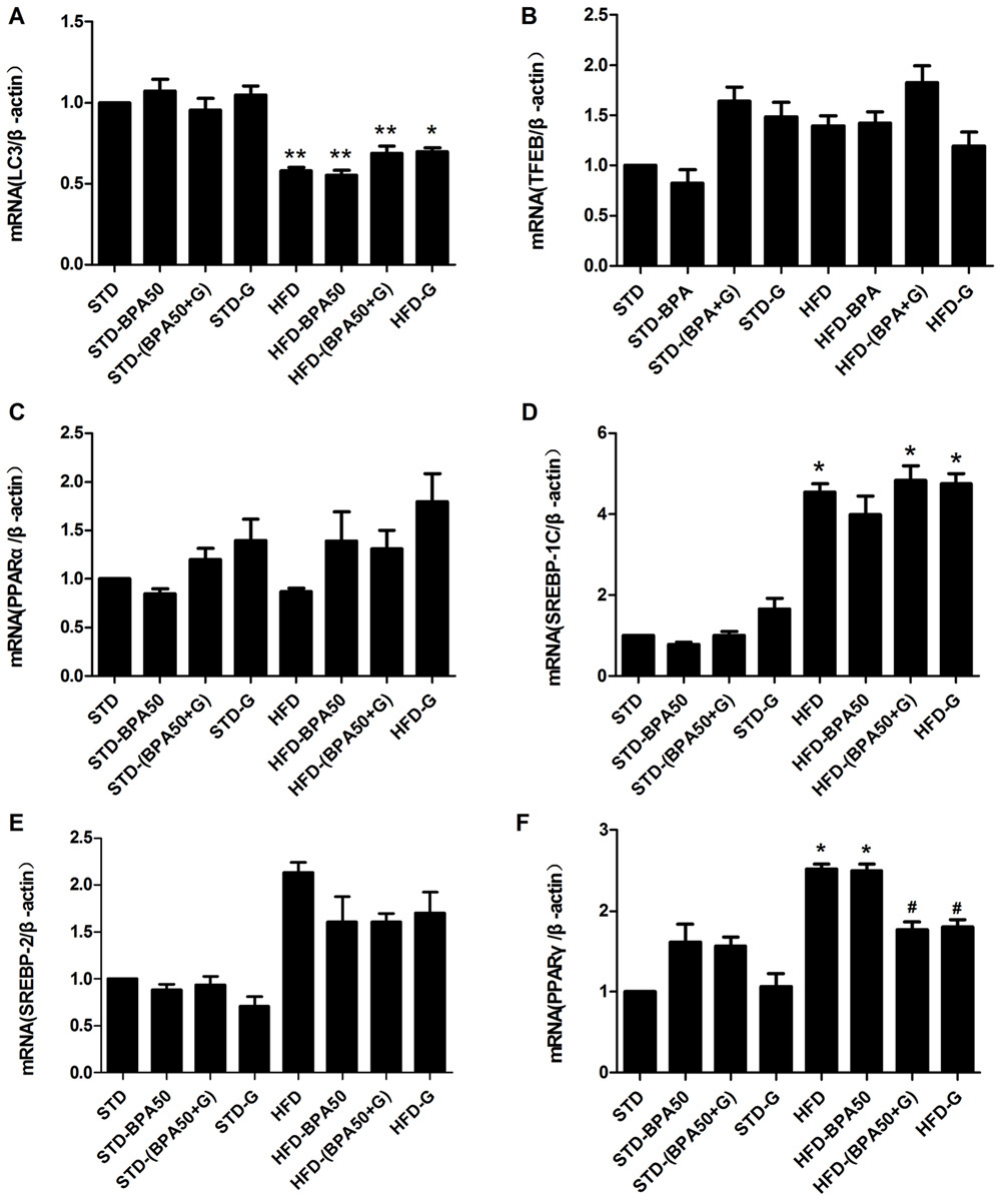
Effect of BPA and genistein on the mRNA expression of liver in rats (n = 3 per group). (A) *LC3*, (B) *TFEB*, (C) *PPARα*, (D) *SREBP1C*, (E) *SREBP2*, (F) *PPARγ* of liver in eight groups. Data are shown as means ± SEM. * *P*<0.05, ** *P*<0.01 compared to STD group; ^#^ *P*<0.05 compared to HFD group.

To determine whether BPA and genistein treatment affected the lipid mechanism of liver, the mRNA levels of *PPARα*, *PPARγ*, *SREBP1C*, *SREBP2*, *TFEB* and *LC3* were determined by quantitative real-time RT-PCR. As shown in Fig 5, HFD markedly increased the mRNA expression of *PPARγ* and *SREBP1C*; and decreased the mRNA expression of *LC3*. Compared to HFD group, the mRNA expression of *PPARγ* was decreased in HFD-(BPA+G) group (*P*<0.05) and HFD-G group (*P*<0.05). The STD- and HFD-fed rats treated with BPA or genistein or both displayed no effect on the mRNA expression of *PPARα*, *SREBP1C*, *SREBP2*, *TFEB* or *LC3* in liver (*P*>0.05).

### The protein expression of liver in rats

**Fig 6:**
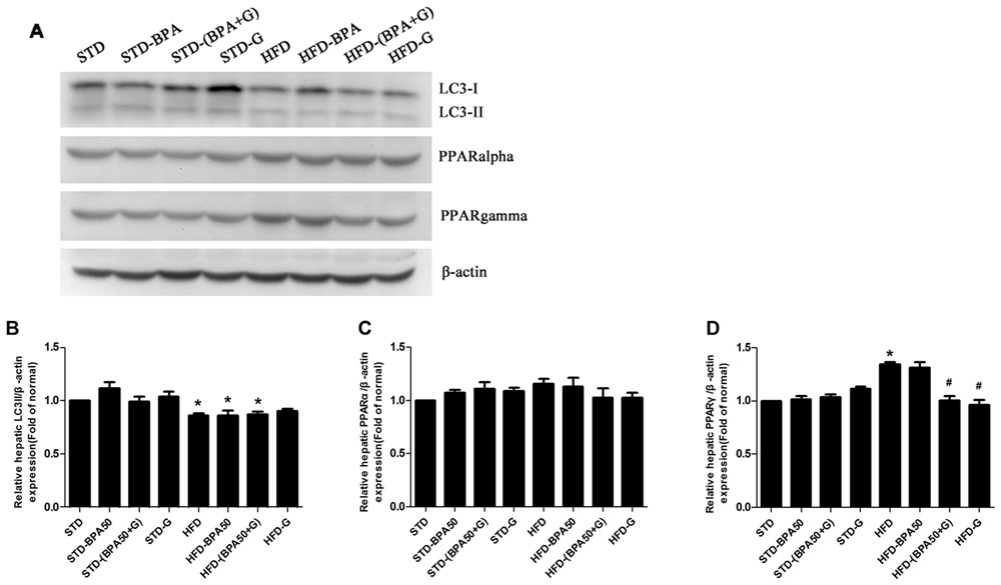
Effect of BPA and genistein on the hepatic protein expression in rats (n = 3 per group). (A) Western blotting results for LC3 PPARα and PPARγ. Relative protein levels of (B) LC3, (C) PPARα and (D) PPARγ. The densities measured by immunoblot analyses were normalized to β-actin were quantitated. Data are presented as mean ± SEM, * *P*<0.05, compared to STD group; ^#^ *P*<0.05 compared to HFD group.

The effects of BPA and genistein on the expression of genes related to hepatic lipid metabolism and marker of autophagy were evaluated by western blot analysis. To confirm whether genistein could regulate hepatic intracellular lipid via activating TFEB and enhancing autophagy, the expression of LC3II (an autophagy marker) and TFEB in liver were examined. (Fig 6B and 6D) illustrates that HFD treatment increased the protein expression of PPARγ (*P*<0.05) and decreased the protein expression of LC3II in liver (*P*<0.05). However, HFD had no effect on the expression of PPARα in liver (*P*>0.05). BPA treatment alone and combined with genistein had no significant effect on the protein expression of LC3II and PPARα in liver of STD- or HFD-fed rats (Fig 6C, *P*>0.05; *P*>0.05). Significant decreasing of the protein expression of PPARγ in liver was observed when genistein was added to rats, compared to either HFD group or HFD-BPA group (Fig 6D).

## Discussion

In our study, we reported that long-term environmental relevant dose of BPA exposure did not affect lipid metabolism in STD- or HFD-fed rats. To mimic the current situation of combined exposure of BPA and genistein on lipid metabolism of populations with Eastern diet and Western diet, we first observed the effects of the co-exposure of BPA and genistein on lipid metabolism in STD- and HFD-fed rats. We demonstrated that long-term genistein treatment alleviated lipid metabolic disorder in HFD-fed adult male rats, this may be due to the regulation of PPARγ (a key gene of lipid metabolism) in liver; and autophagy machinery was not involved in these beneficial effects in STD- or HFD-fed rats. We also observed that the environmental relevant dose of BPA treatment had no synergetic or antagonistic effects on beneficial function of genistein treatment in lipid metabolism.

Our study revealed that the environmental relevant dose of BPA (50μg/kg/day) did not affect general parameters (body weight, total energy intake, liver weight/body weight or subcutaneous body fat percentage). Marmugi *et al*. [9] reported that BPA (5, 50, 500, and 5,000μg/kg/day) did not affect body weight gain or liver weight. However, an increased perigonadic white adipose tissue weight (relative to body weight) was observed at the dose of 50μg/kg/day in six-week-old male CD1 mice. Similarly to our study, Ronn *et al*.[22] reported the insignificant effects of food energy, body weight gain or liver weight/body weight were observed in female Fischer 344 rats treated with 5% fructose and BPA (54.3μg/kg/day and 487.3μg/kg/day, mean). Moreover, BPA (50μg/kg/day) treatment had no effects on lipid parameters (TG and TC in serum or liver) of rats, which was also observed in STD-fed male CD1 mice [9]. Additionally, the environmental relevant dose of BPA exposure had no effect on the histological changes of liver in STD- or HFD-fed rats.

In Western countries and nowadays in China people co-exposed to the environmental relevant dose of BPA, genistein and HFD; and the co-exposed effects of the three factors remains unclear. A prior study *in vivo* revealed that BPA (25 μM) could inhibit glucuronidation of genistein in human and rat liver microsomes [23]. However, until now, whether environmental relevant dose of BPA could disrupt the beneficial effects of genistein is still unclear. In our study, genistein treatment markedly decreased TG and TC levels in serum of HFD-fed rats, and the alteration of TG and TC in liver correlated with changes of serum TG and TC levels. These results indicated that genistein could regulate hepatic lipid metabolism in respond to HFD. Our observations of lipid parameters in rats are also in line with are also in line with Ae Park et al. (2006) in which TG and TC levels were decreased in male C57BL/KsJ-lepr^db^/lepr^db^ (db/db) mice treated continuously with genistein (0.2 g/kg diet) for 6-week [24]. Evidence of improving hepatic lipid accumulation in high-fructose diet fed adult male Wistar rats treated with genistein (1 mg/kg per day) has been reported [25]. A study has reported that STD-fed adult male Wistar rats treated with genistein (1 mg/kg/day) for 60 days had no effect on body weight or liver weight/body weight [25], which is in line with our study. After genistein treatment, we also observed the reducing lipid droplet and lipid accumulating of liver in HFD-fed rats by histological observation in the present study. In our study, no alterations of body weight gain, total energy intake, liver weight/body weight or body fat percentage were observed in STD- or HFD-fed rats after BPA and genistein exposure alone or combined. In addition, BPA treatment also did not induce synergetic or antagonistic effects on liver lipid metabolism in rats treated by genistein.

The classic mechanism (PPARs and SREBPs) and new mechanism (autophagy) on lipid metabolism regulation induced by BPA and genistein were further analyzed in our study. Peroxisome Proliferator-Activated Receptors (α and γ isoforms) are ligand-inducible transcription factors belongs to the nuclear hormone receptor family that play key roles in regulating the expression of genes involved in lipid homeostasis. PPARα has been known as a key regulator of fatty acid oxidation predominantly in mitochondria, peroxisomes, and microsomes of liver [26]. PPARγ is most highly expressed in adipose tissue, which has been demonstrated as a target for the treatment in metabolic diseases and cardiovascular diseases [27,28]. Moreover, PPARγ has a key role in directing critical physiological functions including adipogenesis, glucose and cholesterol metabolism [28]. SREBP1C activates fatty acid synthesis, and SREBP2 regulates cholesterol synthesis and uptake [29]. We investigated the role of BPA and genistein in the modulating of PPARα, PPARγ, SREBP1C and SREBP2 of liver in STD- and HFD-fed rats. There are some *in vitro* studies suggested that halogenated analogs of BPA have the capability to active PPARγ in NIH3T3-L1 cells and human luteinized granulosa cells [30]. And BPA (50 μg/kg) significantly increased the mRNA expression of *SREBP1C* and *SREBP2* of liver in standard diet fed mice [9]. However, no alterations of PPARα, PPARγ, SREBP1C or SREBP2 of liver were observed after the environmental relevant dose of BPA exposure in our study. Moreover, we have shown an ineffective action of genistein treatment on PPARα, SREBP1C and SREBP2 in liver. In the present study, the mRNA and protein expression of PPARγ exhibited significant decreases after genistein treatment. This is consistent with two prior studies observed in rats and mouse. Weigt *et al*. [31] reported that genistein (700 mg/kg diet) treatment for 10-week alleviate the hepatic mRNA expression of *PPARγ* in HFD-fed and ovariectomized Wistar rats. And Jeon *et al*. [31] also confirmed that the hepatic mRNA expression of *PPARγ* was decreased in Male WT mouse fed with HFD containing 0.05% genistein. The possible mechanism for the prevention of NAFLD is as follows: genistein was identified as a PPARγ ligand, which can interact directly with the ligand-binding domain of PPARγ [32]. The activated PPARγ may cause body-wide lipid repartitioning by regulating its target genes, such as P2 (fatty-acid binding protein), CD36 (receptor for lipoproteins), lipoprotein lipase (hydrolysis of lipoproteins), FATP-1 (fatty acid transporter) and SCD-1 (regulators of sterol and fatty acid synthesis, respectively) in visceral adipocyte [33]. Moreover, Kim, *et al*. [34] has also demonstrated that genistein (1, 2 and 4 g/kg diet) may have a preventive effect on NAFLD by the regulation of visceral adipocyte metabolism and adipocytokines. Interestingly, we observed that, contrary to PPARγ expression in adipocyte, PPARγ expression displayed a decreasing in response to genistein in liver of HFD-fed rats. These results indicated that genistein has different regulation on the expression of PPARγ in various tissues. In this study, the down-regulated PPARγ in liver may diminish lipid accumulating via regulating its target genes of downstream; this is corresponding to the enhanced increased PPARγ expression in adipocyte.

Autophagy is a complex and essential process required for the survival and homeostasis of cells. Recently, an increasing number of evidences suggested that both to maintain normal function of liver and to prevent the development of liver disease states (such as NAFLD) is highly dependent on autophagy [35,36]. After a pharmacological or a genetic knockdown inhibition of autophagy, TG content and lipid droplets number and size were increased in hepatocytes under cultured conditions with exogenous lipid supplementation [19]. This indicated that autophagy plays an important role in the cleaning of hepatic lipid droplets accumulation. The transcription factor EB (TFEB) is considered as a master regulator of lysosomal biogenesis and autophagy. It can enter the nucleus and bind to the coordinated lysosomal expression and regulation elements, thereby directly inducing target gene transcription involved in lysosomal biogenesis and autophagy to meet the cellular need [37,38]. Recently, Moskot M *et al* [39] demonstrated that genistein could control TFEB gene expression, TFEB nuclear translocation, and activation of TFEB-dependent lysosome biogenesis to enhance lysosomal metabolism in *in vitro* study. Thus, we studied whether the hepatic TFEB-autophagy pathway is activated by genistein treatment in HFD-fed rats. Compared with STD-fed rats, we observed a decreasing mRNA level of *LC3* in rats fed with HFD for 35-week, in agreement with the decreased amount of LC3II protein and autophagy of liver, which was also observed in male Wistar rats fed with HFD for 10-week [40]. Moreover, BPA and/or genistein treatment had no effects on the expressions of LC3 and TFEB in liver. These findings indicated that genistein may not prevent hepatic lipid accumulating via the regulation of TFEB-autophagy pathway.

Taken together, no adverse effects on lipid metabolism have been observed in either STD-fed or HFD-fed rats exposed to the environmental relevant dose of BPA. Genistein treatment alleviated hepatic lipid metabolic disorder in HFD-fed rats that may be related to down-regulated PPARγ of liver. The environmental relevant dose of BPA treatment did not cause any synergetic or antagonistic effects on genistein’s beneficial roles on hepatic lipid metabolism.

## Supporting Information

S1 Table Body weight data for 35-week. (DOC)

S2 Table Body weight gain data for 35-week. (DOC)

S3 Table Total energy intake data for 35-week. (DOC)

S4 Table Liver weight and body weight ratio data. (DOC)

S5 Table Subcutaneous body fat percentage data. (DOC)

S6 Table Total triglycerides in serum for STD-fed groups. (DOC)

S7 Table Total triglycerides in serum for HFD-fed groups. (DOC)

S8 Table Total cholesterol in serum for STD-fed groups. (DOC)

S9 Table Total cholesterol in serum for HFD-fed groups. (DOC)

S10 Table Hepatic triglycerides data for STD-fed groups. (DOC)

S11 Table Hepatic triglycerides data for HFD-fed groups. (DOC)

S12 Table Hepatic cholesterol data for STD-fed groups. (DOC)

S13 Table Hepatic cholesterol data for HFD-fed groups. (DOC)

## Abbreviations

STD: standard chow diet
HFD: high-fat diet
MetS: metabolic syndrome
NAFLD: non-alcohol fatty liver disease
LC3: microtubule-associated protein 1A/1B-light chain 3
TFEB: the transcription factor EB
PPARα: peroxisome proliferator-activated receptor-alpha
PPARγ: peroxisome proliferator-activated receptor γ
SREBP1C: the sterol regulatory element binding protein 1
SREBP2: the sterol regulatory element binding protein-2
