# Supplementary material for: Environmentally Relevant Dose of Bisphenol A Does Not Affect Lipid Metabolism and Has No Synergetic or Antagonistic Effects on Genistein’s Beneficial Roles on Lipid Metabolism
Source: PLoS One. 2016 May 12;11(5):e0155352. doi: 10.1371/journal.pone.0155352 (PMC4865196; doi:10.1371/journal.pone.0155352)
Supplement: S1 Table — (DOC) [file pone.0155352.s001.doc]

**S1 Table Body weight data for 35-week (Mean)**

| Week | STD | STD-BPA | STD-(BPA+G) | STD-G | HFD | HFD-BPA | HFD-(BPA+G) | HFD-G |
| --- | --- | --- | --- | --- | --- | --- | --- | --- |
| 0 | 199.4 | 195.4 | 191.8 | 196.1 | 197.3 | 193.9 | 196.4 | 195.3 |
| 5 | 409.8 | 408.2 | 398.9 | 410.4 | 461.4 | 470.2 | 481.4 | 478.4 |
| 10 | 486.0 | 506.3 | 490.2 | 499.2 | 542.1 | 551.9 | 552.8 | 558.2 |
| 15 | 542.4 | 550.7 | 538.8 | 547.1 | 610.7 | 618.0 | 605.2 | 620.5 |
| 20 | 565.0 | 572.9 | 560.2 | 569.6 | 623.0 | 636.8 | 633.7 | 650.9 |
| 25 | 609.6 | 616.2 | 604.5 | 615.0 | 660.4 | 674.9 | 670.9 | 671.2 |
| 30 | 611.4 | 610.3 | 598.4 | 616.1 | 668.8 | 682.8 | 681.6 | 681.9 |
| 35 | 609.9 | 622.4 | 605.1 | 619.3 | 665.3 | 666.1 | 668.6 | 656.8 |
