# Supplementary material for: Environmentally Relevant Dose of Bisphenol A Does Not Affect Lipid Metabolism and Has No Synergetic or Antagonistic Effects on Genistein’s Beneficial Roles on Lipid Metabolism
Source: PLoS One. 2016 May 12;11(5):e0155352. doi: 10.1371/journal.pone.0155352 (PMC4865196; doi:10.1371/journal.pone.0155352)
Supplement: S2 Table — (DOC) [file pone.0155352.s002.doc]

**S2 Table Body weight gain data for 35-week**

| **Diet** | **control** | | | **BPA** | | | **BPA+G** | | | **G** | | |
| --- | --- | --- | --- | --- | --- | --- | --- | --- | --- | --- | --- | --- |
|  | mean | SEM | N | mean | SEM | N | mean | SEM | N | mean | SEM | N |
| STD | 396.5 | 18.74 | 10 | 421.5 | 10.72 | 10 | 406.8 | 17.89 | 10 | 413.7 | 14.17 | 10 |
| HFD | 469.1 | 23.91 | 10 | 481.8 | 18.23 | 10 | 468.2 | 22.12 | 10 | 461.5 | 18.13 | 10 |
