# Supplementary material for: Environmentally Relevant Dose of Bisphenol A Does Not Affect Lipid Metabolism and Has No Synergetic or Antagonistic Effects on Genistein’s Beneficial Roles on Lipid Metabolism
Source: PLoS One. 2016 May 12;11(5):e0155352. doi: 10.1371/journal.pone.0155352 (PMC4865196; doi:10.1371/journal.pone.0155352)
Supplement: S3 Table — (DOC) [file pone.0155352.s003.doc]

**S3 Table Total energy intake data for 35-week**

| **Diet** | **Control** | | | **BPA** | | | **BPA+G** | | | **G** | | |
| --- | --- | --- | --- | --- | --- | --- | --- | --- | --- | --- | --- | --- |
|  | mean | SEM | N | mean | SEM | N | mean | SEM | N | mean | SEM | N |
| STD | 84.17 | 1.53 | 5 | 84.25 | 1.34 | 5 | 82.30 | 1.14 | 5 | 80.92 | 1.15 | 5 |
| HFD | 100.97 | 2.06 | 5 | 100.53 | 0.71 | 5 | 105.55 | 3.89 | 5 | 97.62 | 1.87 | 5 |
