# Supplementary material for: Environmentally Relevant Dose of Bisphenol A Does Not Affect Lipid Metabolism and Has No Synergetic or Antagonistic Effects on Genistein’s Beneficial Roles on Lipid Metabolism
Source: PLoS One. 2016 May 12;11(5):e0155352. doi: 10.1371/journal.pone.0155352 (PMC4865196; doi:10.1371/journal.pone.0155352)
Supplement: S4 Table — (DOC) [file pone.0155352.s004.doc]

**S4 Table Liver weight/body weight data**

| **Diet** | **control** | | | **BPA** | | | **BPA+G** | | | **G** | | |
| --- | --- | --- | --- | --- | --- | --- | --- | --- | --- | --- | --- | --- |
|  | mean | SEM | N | mean | SEM | N | mean | SEM | N | mean | SEM | N |
| STD | 2.417 | 0.128 | 10 | 2.586 | 0.104 | 10 | 2.532 | 0.141 | 10 | 2.403 | 0.106 | 10 |
| HFD | 2.707 | 0.175 | 10 | 2.843 | 0.405 | 10 | 2.708 | 0.189 | 10 | 2.422 | 0.118 | 10 |
