# Supplementary material for: Environmentally Relevant Dose of Bisphenol A Does Not Affect Lipid Metabolism and Has No Synergetic or Antagonistic Effects on Genistein’s Beneficial Roles on Lipid Metabolism
Source: PLoS One. 2016 May 12;11(5):e0155352. doi: 10.1371/journal.pone.0155352 (PMC4865196; doi:10.1371/journal.pone.0155352)
Supplement: S5 Table — (DOC) [file pone.0155352.s005.doc]

**S5 Table Subcutaneous body fat percentage data**

| **Diet** | **control** | | | **BPA** | | | **BPA+G** | | | **G** | | |
| --- | --- | --- | --- | --- | --- | --- | --- | --- | --- | --- | --- | --- |
|  | mean | SEM | N | mean | SEM | N | mean | SEM | N | mean | SEM | N |
| STD | 3.51 | 0.21 | 8 | 3.57 | 0.24 | 8 | 3.92 | 0.28 | 8 | 3.40 | 0.16 | 8 |
| HFD | 5.46 | 0.35 | 8 | 6.01 | 0.34 | 8 | 5.97 | 0.40 | 8 | 5.76 | 0.25 | 8 |
