# Supplementary material for: Environmentally Relevant Dose of Bisphenol A Does Not Affect Lipid Metabolism and Has No Synergetic or Antagonistic Effects on Genistein’s Beneficial Roles on Lipid Metabolism
Source: PLoS One. 2016 May 12;11(5):e0155352. doi: 10.1371/journal.pone.0155352 (PMC4865196; doi:10.1371/journal.pone.0155352)
Supplement: S6 Table — (DOC) [file pone.0155352.s006.doc]

**S6 Table Total triglycerides in serum for STD-fed groups**

| **Week** | **control** | | | **BPA** | | | **BPA+G** | | | **G** | | |
| --- | --- | --- | --- | --- | --- | --- | --- | --- | --- | --- | --- | --- |
|  | mean | SEM | N | mean | SEM | N | mean | SEM | N | mean | SEM | N |
| 0 | 0.87 | 0.041 | 10 | 0.82 | 0.034 | 10 | 0.86 | 0.052 | 10 | 0.86 | 0.045 | 10 |
| 21 | 1.04 | 0.031 | 10 | 1.03 | 0.034 | 10 | 1.01 | 0.034 | 10 | 0.97 | 0.028 | 10 |
| 35 | 1.03 | 0.040 | 10 | 1.12 | 0.058 | 10 | 1.05 | 0.053 | 10 | 1.00 | 0.051 | 10 |
