# Supplementary material for: Environmentally Relevant Dose of Bisphenol A Does Not Affect Lipid Metabolism and Has No Synergetic or Antagonistic Effects on Genistein’s Beneficial Roles on Lipid Metabolism
Source: PLoS One. 2016 May 12;11(5):e0155352. doi: 10.1371/journal.pone.0155352 (PMC4865196; doi:10.1371/journal.pone.0155352)
Supplement: S7 Table — (DOC) [file pone.0155352.s007.doc]

**S7 Table Total triglycerides in serum for HFD-fed groups**

| **Week** | **control** | | | **BPA** | | | **BPA+G** | | | **G** | | |
| --- | --- | --- | --- | --- | --- | --- | --- | --- | --- | --- | --- | --- |
|  | mean | SEM | N | mean | SEM | N | mean | SEM | N | mean | SEM | N |
| 0 | 0.83 | 0.043 | 10 | 0.86 | 0.043 | 10 | 0.83 | 0.040 | 10 | 0.83 | 0.031 | 10 |
| 21 | 1.50 | 0.042 | 10 | 1.52 | 0.032 | 10 | 1.50 | 0.047 | 10 | 1.48 | 0.036 | 10 |
| 35 | 1.63 | 0.039 | 10 | 1.71 | 0.035 | 10 | 1.64 | 0.030 | 10 | 1.60 | 0.032 | 10 |
