# Supplementary material for: Environmentally Relevant Dose of Bisphenol A Does Not Affect Lipid Metabolism and Has No Synergetic or Antagonistic Effects on Genistein’s Beneficial Roles on Lipid Metabolism
Source: PLoS One. 2016 May 12;11(5):e0155352. doi: 10.1371/journal.pone.0155352 (PMC4865196; doi:10.1371/journal.pone.0155352)
Supplement: S8 Table — (DOC) [file pone.0155352.s008.doc]

**S8 Table Total cholesterol in serum for STD-fed groups**

| **Week** | **control** | | | **BPA** | | | **BPA+G** | | | **G** | | |
| --- | --- | --- | --- | --- | --- | --- | --- | --- | --- | --- | --- | --- |
|  | mean | SEM | N | mean | SEM | N | mean | SEM | N | mean | SEM | N |
| 0 | 1.91 | 0.042 | 10 | 1.95 | 0.033 | 10 | 1.89 | 0.036 | 10 | 1.96 | 0.023 | 10 |
| 21 | 2.00 | 0.062 | 10 | 2.12 | 0.072 | 10 | 2.00 | 0.100 | 10 | 2.20 | 0.060 | 10 |
| 35 | 2.91 | 0.106 | 10 | 2.94 | 0.067 | 10 | 3.00 | 0.084 | 10 | 3.07 | 0.060 | 10 |
