# Supplementary material for: Environmentally Relevant Dose of Bisphenol A Does Not Affect Lipid Metabolism and Has No Synergetic or Antagonistic Effects on Genistein’s Beneficial Roles on Lipid Metabolism
Source: PLoS One. 2016 May 12;11(5):e0155352. doi: 10.1371/journal.pone.0155352 (PMC4865196; doi:10.1371/journal.pone.0155352)
Supplement: S9 Table — (DOC) [file pone.0155352.s009.doc]

**S9 Table Total cholesterol in serum for HFD-groups**

| **Week** | **control** | | | **BPA** | | | **BPA+G** | | | **G** | | |
| --- | --- | --- | --- | --- | --- | --- | --- | --- | --- | --- | --- | --- |
|  | mean | SEM | N | mean | SEM | N | mean | SEM | N | mean | SEM | N |
| 0 | 1.95 | 0.029 | 10 | 2.01 | 0.039 | 10 | 1.96 | 0.039 | 10 | 1.96 | 0.023 | 10 |
| 21 | 2.76 | 0.047 | 10 | 2.84 | 0.150 | 10 | 2.42 | 0.150 | 10 | 2.42 | 0.040 | 10 |
| 35 | 3.38 | 0.132 | 10 | 3.02 | 0.157 | 10 | 2.93 | 0.157 | 10 | 2.93 | 0.138 | 10 |
