# Supplementary material for: Environmentally Relevant Dose of Bisphenol A Does Not Affect Lipid Metabolism and Has No Synergetic or Antagonistic Effects on Genistein’s Beneficial Roles on Lipid Metabolism
Source: PLoS One. 2016 May 12;11(5):e0155352. doi: 10.1371/journal.pone.0155352 (PMC4865196; doi:10.1371/journal.pone.0155352)
Supplement: S10 Table — (DOC) [file pone.0155352.s010.doc]

**S10 Table Hepatic triglycerides data for STD-fed groups**

| **Week** | **control** | | | **BPA** | | | **BPA+G** | | | **G** | | |
| --- | --- | --- | --- | --- | --- | --- | --- | --- | --- | --- | --- | --- |
|  | mean | SEM | N | mean | SEM | N | mean | SEM | N | mean | SEM | N |
| 35 | 15.15 | 0.45 | 8 | 15.00 | 0.89 | 8 | 16.00 | 0.69 | 10 | 16.00 | 0.72 | 10 |
