# Supplementary material for: Environmentally Relevant Dose of Bisphenol A Does Not Affect Lipid Metabolism and Has No Synergetic or Antagonistic Effects on Genistein’s Beneficial Roles on Lipid Metabolism
Source: PLoS One. 2016 May 12;11(5):e0155352. doi: 10.1371/journal.pone.0155352 (PMC4865196; doi:10.1371/journal.pone.0155352)
Supplement: S11 Table — (DOC) [file pone.0155352.s011.doc]

**S11 Table Hepatic triglycerides data for HFD-fed groups**

| **Week** | **control** | | | **BPA** | | | **BPA+G** | | | **G** | | |
| --- | --- | --- | --- | --- | --- | --- | --- | --- | --- | --- | --- | --- |
|  | mean | SEM | N | mean | SEM | N | mean | SEM | N | mean | SEM | N |
| 35 | 21.17 | 0.72 | 8 | 20.49 | 0.83 | 8 | 18.37 | 0.46 | 10 | 18.16 | 0.44 | 10 |
