# Supplementary material for: Environmentally Relevant Dose of Bisphenol A Does Not Affect Lipid Metabolism and Has No Synergetic or Antagonistic Effects on Genistein’s Beneficial Roles on Lipid Metabolism
Source: PLoS One. 2016 May 12;11(5):e0155352. doi: 10.1371/journal.pone.0155352 (PMC4865196; doi:10.1371/journal.pone.0155352)
Supplement: S13 Table — (DOC) [file pone.0155352.s013.doc]

**S13 Table Hepatic cholesterol data for HFD-fed groups**

| **Week** | **control** | | | **BPA** | | | **BPA+G** | | | **G** | | |
| --- | --- | --- | --- | --- | --- | --- | --- | --- | --- | --- | --- | --- |
|  | mean | SEM | N | mean | SEM | N | mean | SEM | N | mean | SEM | N |
| 35 | 7.07 | 0.25 | 8 | 7.25 | 0.14 | 8 | 6.24 | 0.11 | 10 | 6.19 | 0.09 | 10 |
